# Superabsorbent Polymers for Internal Curing Concrete: An Additional Review on Characteristics, Effects, and Applications

**DOI:** 10.3390/ma17225462

**Published:** 2024-11-08

**Authors:** Bo Zhou, Kejin Wang, Peter C. Taylor, Yucun Gu

**Affiliations:** 1 Department of Civil, Construction and Environmental Engineering, Iowa State University, Ames, IA 50011, USA; bozhou@iastate.edu (B.Z.); kejinw@iastate.edu (K.W.); 2 National Concrete Pavement Technology Center, Iowa State University, 2711 South Loop Drive, Ames, IA 50010, USA; 3 Department of Civil, Architectural and Environmental Engineering, Missouri University of Science and Technology, Rolla, MO 65401, USA; gywvy@mst.edu

**Keywords:** SAP, internal curing, concrete mixtures, workability, durability

## Abstract

Superabsorbent polymers (SAPs) are a promising admixture that can provide internal curing to freshly cast concrete and enhance concrete properties. Although many reviews have explored aspects of SAPs, the links among SAPs’ chemical and physical properties, internal curing behaviors, concrete performance, and their large-scale applications are often weakly elucidated. This paper provides an additional review of the chemical structures and physical dimensions of SAPs and their effects on the internal curing kinetic behavior as well as on concrete properties, such as workability, strength, and durability. In addition, different approaches to introducing SAP particles into concrete mixtures are also summarized. Case studies on the use of SAPs in the concrete industry are introduced to provide a better understanding of the greatest potential of SAPs in field applications. The results confirm that the utilization of SAPs in concrete mixtures provides multiple benefits such as improved water curing, reduced shrinkage, and enhanced workability. Selecting the appropriate SAPs is crucial and involves considering factors like absorption rate, durability, and stability. However, achieving uniform distribution of dry SAPs in concrete poses challenges. Further research is required to gain a deeper understanding of the impact of SAPs on transport properties and frost durability. Additionally, the absence of a standard makes it difficult to maintain consistent water-to-cement ratios. These findings provide a theoretical foundation for using SAPs to enhance concrete performance while also highlighting future research directions and challenges. In this article, scientists, engineers, and contractors will find a comprehensive explanation encompassing laboratory investigations, field implementation, and relevant guidance.

## 1. Introduction

Water in a cementitious system can be lost through two mechanisms: (1) consumption by cementitious hydration and (2) evaporation due to drying. In either case, the effects lead to shrinkage and cessation of cementitious hydration. The use of internal curing (IC) agents, such as lightweight aggregates (LWA) and superabsorbent polymers (SAP), is beneficial for concrete structures because of their porous nature, which allows them to absorb and hold moisture within the concrete matrix in times of high moisture content. Moreover, they can release stored moisture to surrounding cement paste when moisture levels decrease, preventing detrimental effects of drying out [1,2,3].

In recent decades, IC has been demonstrated to provide significant benefits in concrete, particularly in high-performance concrete (HPC), where the water-to-binder ratio (w/b) is low (≤0.42). Because internal water makes concrete less prone to self-desiccation and drying, internally cured concrete commonly demonstrates reduced autogenous shrinkage and drying shrinkage [4,5,6]. As cement hydration continues to result from the availability of internal water, internally cured concrete often exhibits improved mechanical properties, durability, and extended service life.

Presently, LWA has been widely accepted as an effective IC agent in the USA. SAPs with hydrophilic structures can absorb and retain water and aqueous solutions thousands of times their dry weight without dissolving, and they show superior desorption in comparison to other candidate IC materials [7,8]. SAP can absorb and release water more effectively during hydration without requiring pre-saturation. In contrast, LWA must be pre-wetted before use. This desorption behavior allows SAPs to gradually release stored water over time, promoting continuous hydration and reducing shrinkage in cement-based materials. Furthermore, SAPs are highly efficient in controlling the internal moisture distribution, ensuring that the microstructure of concrete remains more intact and durable [9,10].

However, the IC effectiveness of SAPs largely depends on their intrinsic characteristics, including the mechanical response, swelling response, shape, and size of the SAP hydrogels. These parameters directly affect their ability to retain and release water in response to the concrete’s hydration demands, playing a critical role in mitigating shrinkage and enhancing durability [11]. Recently, several review articles have been published on SAPs, offering insight into various facets of these materials. Some reviews have focused on the production methods of SAPs, which range from free-radical polymerization to controlled radical techniques. Each method influences the final polymer’s molecular structure and functional properties [12,13,14]. Other research efforts have classified SAPs based on their chemical composition, structure, and functionality, linking these characteristics to a wide array of applications, including biomedical uses, environmental, and industrial sectors [15,16]. Notably, SAPs have been extensively applied in agriculture, where they help retain soil moisture and improve nutrient delivery, offering substantial benefits in arid regions [17,18,19]. Moreover, a growing body of literature has investigated their role in cement-based composite applications, showing their ability to improve workability, reduce autogenous shrinkage, and enhance the mechanical properties of concrete [7,20,21,22]. However, there is limited literature that provides a comprehensive analysis of the intrinsic properties of SAPs and their effects on concrete performance, as well as relevant standards and practical applications.

Therefore, this review aims to evaluate the role of SAPs in enhancing concrete performance through internal curing, focusing on the connections between SAP characteristics—such as absorption rate, particle size, and crosslinking density—and their effects on concrete properties like shrinkage reduction, workability, and durability. Additionally, there is a noticeable gap in addressing relevant standards and practical guidelines for applying SAPs in large-scale concrete structures. As the construction industry increasingly adopts SAPs for improved performance, a thorough analysis that bridges the gap between research and practical implementation is essential. This analysis will provide clearer insights into optimizing SAPs for specific concrete applications, ensuring consistent results across various projects.

## 2. Concept of Internal Curing

### 2.1. Definition of Internal Curing

Concrete is a nonuniform composite material comprising combinations of aggregates and cementitious paste [23]. When a cementitious material is mixed with water, the active components like C_3_S will hydrate to form crystalline and gel-like hydration products, which interact physically causing the mixture to set and gain strength. If water is lost significantly due to evaporation and/or desiccation at an early stage, cement hydration will decrease. This prevents the development of concrete strength and impermeability [24,25,26]. At the same time, concrete begins to shrink and generates stresses, increasing the risk of cracking [27,28]. That is, water plays a key role in the formation of concrete microstructure and the development of concrete properties.

**Figure 1:**
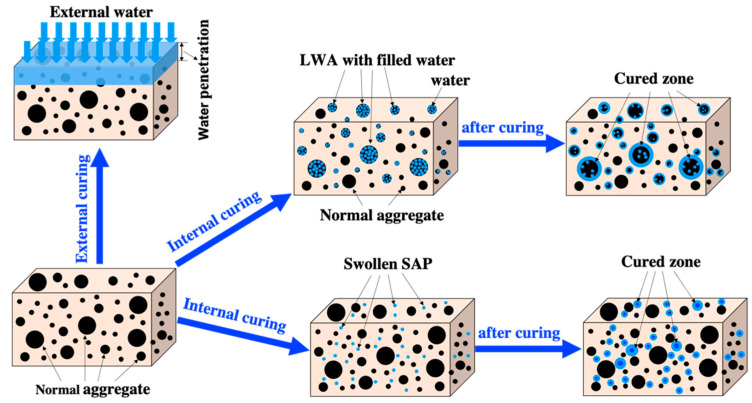
External and internal curing of the specimen.

Curing methods, including external curing and internal curing, are utilized to minimize water loss, as shown in Figure 1. Conventional concrete surface curing methods include sprinkling extra water or covering it with burlap and plastic sheets to reduce water loss. However, these methods do not address water loss due to internal desiccation. In addition, externally supplied water will only penetrate a limited distance from the surface (see Figure 1). Internally stored water, on the other hand, can benefit the full depth of the concrete section, which ensures better hydration and shrinkage mitigation, particularly in high-performance concrete.

IC can be achieved by the judicious use of absorbent materials such as expanded shale, clay, fly ash, rice husk ash, LWA, and SAP [29].

Benefits of internal curing include (1) an increased degree of hydration (and so better mechanical and durability properties); (2) reduced shrinkage (and so lower cracking risk); and (3) reduced moisture differentials (and so reduced warping in thin slabs).

### 2.2. Required Properties of an IC Agents in Concrete

IC requires the use of materials able to freely release the stored water to the cement paste after setting, which implies that any potential IC medium must comply with two requirements. The first one is that water stored in the IC medium should have an equilibrium RH of 99% when the meniscus diameter is 100 nm; in other words, it has thermodynamic availability [30].

Another one is that it should have a kinetic availability to transfer the water to the cement paste as needed when the RH in the surrounding paste matrix drops. In addition, the stored curing water should be uniformly spatially distributed to reduce the internal stresses due to differential drying. This can be improved by decreasing the size of the water-containing pores and increasing the number of pores in the IC medium [29,31].

## 3. Basic Physical and Chemical Properties of SAP

SAP is a functional macromolecule with a three-dimensional network structure that has exceptional water absorption and retention abilities. The use of these products in hygiene and agriculture has been extensively researched and is widely used due to their properties. SAPs are commonly synthesized from acrylic acid and derivatives, undergoing crosslinking reactions to create a highly absorbent structure. However, not all ready-made SAP is suitable and beneficial for cementitious building materials, as the majority of synthesized SAP products primarily focus on performance in other industrial applications such as the hygiene industry producing diapers or drug delivery systems [32]. Hansen and Jensen introduced these polymeric materials as a water-entraining additive in construction materials technology [33].

The properties desired for an SAP to be effective in concrete systems include the following:

- Ability to rapidly absorb sufficient water during mixing.
- Ability to retain integrity during mixing.
- Ability to desorb when required (i.e., when RH drops below ~94%).
- Appropriate particle size distribution.

### 3.1. Absorption and Desorption

The quantity of liquid absorbed, absorption kinetics and potential desorption, and stability of the product formed in solution depend on thermodynamics and the osmotic pressure differences between the gel network and the exterior environment [34].

Different from the addition of pre-wetted LWA into the cementitious system, dry SAP can be mixed with raw concrete materials before water is added during concrete mixing, thus helping the uniform dispersion of SAP. Therefore, the SAP has to absorb the right amount of water from the mixture while it is still being mixed and transported. As a result, the desorption rate of SAP is a significant factor as it determines the timing and amount of water supplied to the paste [35].

**Figure 2:**
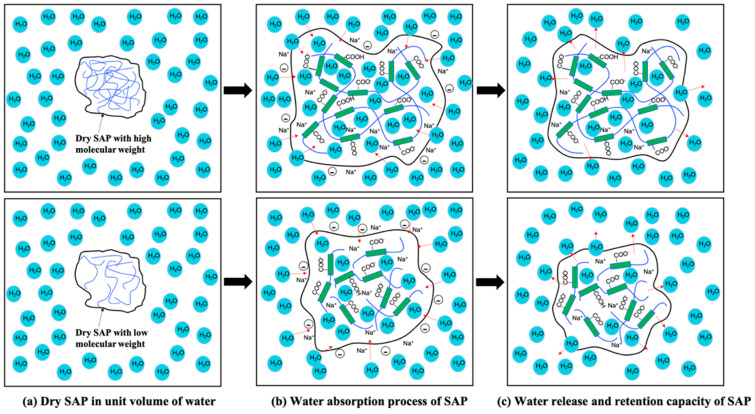
Absorption and desorption behaviors of SAP with high/low molecular weights.

It is well known [36,37,38] that absorption and desorption kinetics are closely related to crosslinking density, molecular structure (see Figure 2), and particle size of SAP. The crosslinking density of SAPs is crucial in determining their mechanical properties and water retention capacity. A higher crosslinking density creates a more rigid network, improving the material’s strength but reducing its swelling capacity. This directly impacts concrete performance by limiting the available curing water. On the other hand, a lower crosslinking density allows for more swelling and higher water retention but may weaken the SAP’s mechanical properties, making it less durable during mixing and transportation in concrete applications [39,40,41].

When in contact with water, high molecular weight SAPs ionize with hydrophilic groups such as carboxylic and hydroxyl groups. As shown in Figure 2a, dry SAP has a three-dimensional (3D) network structure made up of tangled molecular chains. The molecular structure of SAP directly influences its water retention and desorption, critical for minimizing concrete shrinkage. Higher molecular weight and lower crosslinking density provide greater internal space, allowing for more water retention. As the concrete dries, the water is gradually released, promoting hydration, reducing shrinkage, and enhancing the concrete durability [3,42,43]. When the hydrophilic groups in the polymer chain get ionized, a difference in ionic concentration will arise between the inside and outside of the molecule. This will lead to the formation of gel caused by successive ingress of water molecules into the internal structure. SAP containing different molecular weights exhibits quite different molecular bond expansion. Therefore, SAP with higher molecular weight would have more internal network space, which corresponds to an enhanced retention capacity due to the migration of anions and cations. However, when the release of water from SAP occurs, the expansion of molecular bonds would also be influenced by the molecular weight of SAP. Molecular bonds are restricted in their expansion as SAP’s molecular weight increases. This is attributed to the weak bonding between the water molecule and hydrophilic groups in the molecular chain structure of SAP. For example, SAP with a smaller molecular weight has less 3D space formed to store water molecules after the expansion of the molecular chain. This in turn results in the water molecules absorbed by the hydrophilic groups not being able to escape easily. Therefore, the release of free water from the molecular chain structure is slower, as shown in Figure 2b,c.

In Figure 2, the kinetic behavior of SAP absorption and desorption in concrete provides insight into the physicochemical properties of the polymers added to the concrete. Time-resolved experiments on swelling must also consider the rate of absorption since it is considered one factor of assessment throughout the interval between mixing and placing concrete. After the final placement, no further water needs to be absorbed as the framework for the system is locked in. Hence, SAP’s sorption properties determine how SAP will affect concrete’s properties both in the short- and long-term. Its short-term effects include affecting water absorption during mixing, contributing to internal curing, and influencing early strength development. In the long term, SAPs contribute to concrete’s chemical stability, mitigate drying shrinkage, and enhance overall durability. An exploration of the SAP’s sorption properties in engineering applications holds the potential to yield valuable insights, guiding improvements in concrete formulations and enhancing workability over time. The discussion on the impacts of SAPs on concrete performance will be presented in subsequent sections.

### 3.2. Physical/Chemical Stability

SAP as an internal curing agent in concrete should have sufficient physical stability to remain as a whole during the rigors of mixing, transportation, and placing [44]. Generally, the mechanical strength of SAP is correlated with the cross-linking density inside its gel structure. When SAP has a higher cross-linking density, its gel strength becomes higher [44]. However, to some extent, this will reduce the swelling capacity of the gel sample. Therefore, some researchers [45,46,47] have proposed strategies to improve it by using double-networking and nanoparticles, which makes the gel sample have both good water absorbency and mechanical properties. For example, Ma et al. [48] studied ethylene glycol diglycidyl ether as a surface cross-linking agent to enhance the surface cross-linking of SAP particles, and the results showed that this method was effective at improving the gel strength of SAP. This equilibrium is pivotal as it directly affects the subsequent pore size after SAP releases water, which will be introduced in the next section.

Furthermore, the sustained functionality of SAP in concentrated cementitious pore solutions is crucial. Exposure to various ionic solutions (Na^+^, Ca^2+^, and Al^3+^) should not compromise the chemical bonds within the SAP microstructure, ensuring stability in diverse environments.

### 3.3. Particle Sizes

The mechanical performance of concrete paste is strongly influenced by the size and amount number of voids in the hardened system. It is preferable to have a large number of small voids than a few large ones that will act as areas of stress concentration. As mentioned earlier, in order to achieve a uniform distribution of curing water, it is preferable to have a greater number of small voids rather than a few large ones. It’s important to note that when the SAP particles release their water, they will create voids.

In addition, the maximum absorption capacity and absorption rate are influenced by the particle size of SAP. The smaller the particle size of the SAP particles, the larger the exposure surface and the smaller the distance that water needs to travel to fully saturate the particle. Luís Pedro Esteves [49] used Fick’s second law of diffusion to show the nonlinear relationship between the absorption property of SAP with its particle size (diameter):

(1) $$\frac{d_{Q}}{d_{t}}=k*\left(Q_{max}-Q\right)$$

where Q_max_ and Q are the swelling capacities at equilibrium and at any time *t*, and *k* is the swelling constant rate, which depends on the particle size.

A solution/bulk polymerization technique is currently used to synthesize SAPs, a cross-linking bulk of randomly composed copolymer obtained with this technique needs to be broken into fine particles to meet this requirement. Consequently, an irregular shape of SAP particles is obtained, and their particle sizes are related to the selection mechanism [50,51]. If inverse suspension polymerization is utilized, the SAP will be spherical, with a narrow size distribution [16,50]. Various particle sizes of dry SAPs have been used in cementitious materials typically below 100 μm [29,51,52,53,54,55].

### 3.4. Chemical Characteristics of SAP

**Figure 3:**
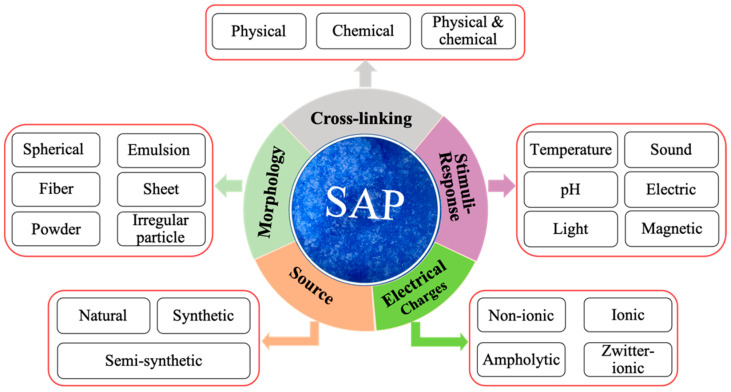
Classification of SAP based on the different characteristics.

The potential efficiency of SAPs as IC agents in practical applications is affected by the swelling properties, sorption kinetics, type, dosage, and particle size of the SAPs. Additionally, the cross-linking density/degree is closely correlated with swelling behaviors and sorption kinetics of SAP. The classification of SAP is based on morphology, chemical building blocks, cross-linking mechanism, and the types of electrical charges present [56], as shown in Figure 3.

There are two main kinds of crosslinks in the chains of most SAP, namely bulk or core crosslinking and surface crosslinking. The former occurs in the polymerization process of SAP production and is governed by their reactivity ratios [57]. Normally, the lower the cross-linking level, the higher the swelling capacity of the polymer. The latter can be modified in the post-process to improve the swelling rate and the resistance to pressure absorption of SAP, which contributes to maintaining the original shape of particles when immersed. Cross-linking of SAPs includes both physical cross-linking and chemical cross-linking. The weak physical bonds like hydrogen bonds in the physical cross-linking chain are unstable whereas the chemical covalent bonds are stable and strong.

Products are also classified based on the presence of electrical charges in the side chains of molecules as ionic (charged) and non-ionic (neutral). Currently, commercially available SAPs are mostly ionic polymers.

Most SAPs used for IC are polyacrylate acids (PAA) and polyacrylamides (PAM), both containing amide groups that can react with water to form carboxylate groups with hydrogen. These can be deprotonated, leaving anionic charges left along the side chains of SAPs. The polyacrylic acids are the most used commercial polymers, made by using the suspension polymerization technique. Moreover, these SAPs with high molecular weights (up to a few million g/mol) show a higher absorption capacity compared to other types of SAPs. However, the absorption capacity of these commercial SAPs for application in concrete systems is reduced due to the presence of cations and anions in the cement paste. PAA-based SAPs have higher water absorption capacities, which can lead to improved internal curing and early hydration. However, this may also result in lower strength at later stages due to prolonged water retention. On the other hand, PAM-based SAPs, with lower absorption capacities, release water more slowly, aiding in better shrinkage control and enhancing long-term durability and strength. When combined with materials such as metakaolin, SAPs can further improve concrete performance by enhancing hydration, reducing shrinkage, and strengthening the material based on the specific SAP used [58,59,60].

It is necessary to understand the sorption kinetics between SAP and both water and pore fluid because the properties of SAP are affected by solution chemistry [61,62]. Therefore, the reaction variables forming the SAP must be optimized to obtain a suitable balance among properties based on the target application. It should achieve a stable thermodynamic equilibrium (swelling equilibrium at the balance of osmotic pressure), as shown in Equation (2) [63]:

(2) $$∆\pi =∆\pi_{elastic}+∆\pi_{mix}+∆\pi_{ion}+∆\pi_{bath}=0$$

where Δπ is the swelling equilibrium pressure of the hydrogel, Δπ_elastic_ is an elastic force exerted by the polymer network, Δπ_mix_ is the osmotic pressures generated by the polymer-solvent interactions during the mixing phase, Δπ_ion_ is the expansive pressure contributed by the electrostatic interactions of polyelectrolyte hydrogels, and Δπ_bath_ is the contribution of ionic strength from the surrounding bath solution.

In essence, most SAPs are a cross-linked structural polymer with a three-dimensional network. The water molecules will first penetrate the cross-linked network, resulting in a difference in osmotic pressure Δπ_mix_ between outside and inside the polymer network. On the other hand, due to the interactions between polymers, the primary polymer network in the chain is limited to moving freely; therefore, an elastic force Δπ_elastic_ counteracts the expansive force.

Polyelectrolyte hydrogels consist of weak acidic and weak basic groups, respectively, which cause the hydrophilic group in the chain of SAP to ionize, yielding a difference in ion concentration between the internal solutions. When acrylic-based SAP is used in a cementitious environment, the hydrogel with acidic groups will be almost completely deprotonated; for example, almost all carboxylic groups have negative charges [64]. Therefore, the neighboring chains with likewise charged groups developed electrostatic repulsion Δπ_ion_ between chains, which contributes to the balance of the osmotic pressure [65]. The hydrogel system, however, must maintain electrical neutrality within itself, therefore the counterions cannot be released from the system unless they’re replaced by another mobile ion from the surrounding environment. If the surrounding environment of SAP is the cement-based medium, the enriched calcium ions Ca^2+^ can exchange with the mobile ions of the gel in the SAP network, which will yield the osmotic pressure due to the different ion concentrations inside and outside of the polymer network structure. For example, the results from Schröfl et al. [35,66] verified that the presence of Ca^2+^ reduced the retention capacity of the SAP, but it still counteracted autogenous shrinkage during the acceleration period of cement hydration.

## 4. Effects of SAPS on Concrete Properties

### 4.1. Batching SAPs

Different approaches to introducing particles into concrete mixtures have been studied. Each approach leads to different influences on the development of concrete properties. Table 1 lists the effects of different batching processes on concrete performance.

#### 4.1.1. Presoaking

When LWFA is used for internal curing, it is practiced by presoaking the aggregate before batching [76]. This can lead to challenges in high-volume construction sites.

However, the absorption properties of SAP are quite different because the sorption of SAP is a physicochemical process that is more efficient than the purely physical process in LWA. SAP particle sizes are often smaller with a higher absorption rate.

SAP particles are presoaked for different periods based on the corresponding requirement for the water content prior to mixing, then the presoaked SAP particles are mixed with other concrete mixtures. However, there is no standard guidance for the sequence of presoaked SAP particles added to the concrete mixtures as shown in Table 1.

The influence of pre-saturated SAP as an IC medium on the workability, early-age shrinkage, and mechanical properties of concrete was conducted by Azari et al. and Jafari et al. [52,70]. Their results revealed that the incorporation of pre-soaked SAP could not only improve the static stability and passing ability of fresh concrete but mitigate the shrinkage at an early age, corresponding to alleviating the risk of shrinkage cracking, which was attributed to the compensation of water loss from the release of SAP. Additionally, a slight decrease in compressive strength was also reported due to the addition of pre-soaked SAP.

The data in Table 2 demonstrate the effects of different ways of adding SAP into concrete mixtures, including pre-soaked or dry SAPs, and their specific impact on workability, stability, and shrinkage control. Pre-soaked SAP enhances the flowability and reduces the static stability of fresh concrete mixtures. However, it also causes a slight decrease in compressive strength at early stages. Table 2 suggests that pre-soaking SAP is more effective in controlling early-age shrinkage and improving static stability, but it presents challenges in controlling water release during mixing. It indicates that optimizing the timing and method of SAP addition is crucial for maintaining concrete workability and ensuring even moisture distribution.

#### 4.1.2. Dry Batching

Much research work has been conducted investigating dry SAP batching. It has been verified that this approach can distribute the SAP particles without agglomeration [77,78,79]. Attention has been paid to how the absorption rate of dry SAP affects the mixing and handling processes while achieving a suitable state of saturation. Viktor et al. [80] demonstrated that absorption of 8–30 g/g of the SAP was desirable for use in a highly alkaline solution, which is about 10 times less than in a tap water solution.

The water absorption and release processes of SAPs in fresh concrete are influenced by whether the dry or pre-wetted SAPs are used. It is noteworthy that dry SAP takes in water rapidly and substantially, particularly distilled water. This results in a decreased water-binder ratio in the mixing process, consequently reducing the workability of the concrete mixture. On the contrary, the pre-wetted SAP may release less water. This subtle variation in water release plays a significant role in regulating the rise of the effective water-binder ratio. These insights are essential for optimizing concrete mix designs and achieving the desired equilibrium between water absorption, release, and overall concrete performance. In summary, during the preparation of a concrete mixture, the addition of pre-soaked SAP is more efficient at enhancing workability compared to the use of dry SAP, whereas the latter method can enhance uniform dispersion of the polymer [38].

### 4.2. Workability

When SAP is used in concrete, the effective water demand must be considered because of its effect on mixture workability. Jensen et al. [35,81] studied an entrained effective water-to-cement of 0.05 while a certain kind of SAP content of 0.4 wt.% was used. The results showed that adding SAP to the mixture would reduce the workability if additional water was not added. To achieve the same target comparable workability in SAP-cured concrete mixtures and control mixtures, the methods of introducing additional water or superplasticizers have been employed by researchers [25,82]. The amount of extra water integrated into the cementitious materials correlates with the water-to-cementitious material ratio (w/cm), impacting the workability of the fresh concrete mixture. This incorporated extra curing water serves a dual purpose, providing sufficient moisture for the cementitious matrix to improve its durability, particularly in mitigating shrinkage effectively. Simultaneously, it helps facilitate the desired flow and placement of the mixture during construction. Currently, the methods for determining the addition of extra water content rely on the Power’s model [33], or capillary absorption [83], or water sorption kinetics of SAP [84,85]. However, these methods for the calculation of extra water content have limitations in predicting concrete workability. Assessing the hydration degree of cementitious composite material based on theoretical equations like Power’s model proves challenging. Assmann’s capillary method may not be well-suited for concrete application, potentially due to the interference of aggregates and setting time constraints. Similarly, the prevalent methods, such as the teabag method and filtration method, involving the internal curing water content absorbed by SAP are influenced by solid phases or mineral admixtures like silica fume in concrete mixtures [4,86]. Consequently, a precise, widely applicable method for evaluating the incorporation of extra water content in concrete mixtures needs further development to ensure the satisfactory workability of SAP-modified concrete.

Furthermore, the effects of SAP on the workability of concrete correlate with factors such as particle sizes, crosslinking density, and water absorption rates (depending on whether the SAP is dry or presoaked). Large particles may increase water absorption, reducing hydration reactions and stiffening the concrete mixture. Conversely, smaller particles can enhance workability by facilitating better distribution and dispersion within the concrete matrix. For example, Azarijafari et al. [52] showed that finer SAP could be more effective in improving workability than coarser SAP. Additionally, the workability of concrete is affected by the crosslinking density. A higher crosslinking density reduces the polymer’s ability to swell and absorb water, as the tightly bound network restricts expansion. Conversely, a lower crosslinking density allows for greater water retention, as the SAP network can swell more easily. This balance is crucial in its applications in concrete, where optimal water retention is essential for hydration and durability [43]. Water absorption rates also play a crucial role in influencing workability. For example, Dang et al. [69] conducted a comparison of the impact of dry and presoaked SAP on the workability of concrete. Results showed that as expected the use of prewetted SAP caused an increase in concrete slump, while dry SAP resulted in a reduction in concrete workability and a delay of setting time [52,69,87].

Although some studies have shown that the cement hydration process influences the water release process of SAP, there is a need to correlate the absorption rate of SAPs with changes in the workability of fresh concrete. The reason for this is that the absorbency rate of SAP can impact fresh concrete’s workability. As SAP absorbs water from a mixture, less water is available for hydration reactions, making the concrete stiffer and less workable. In addition, SAP produces gel-like structures that influence water distribution within mixtures, further impacting workability. Considering specific SAP characteristics and concrete mix design, it is necessary to carry out experimental studies to correlate SAP absorption rate with changes in workability.

### 4.3. Strength

Several studies have investigated the impacts of SAP on concrete mechanical properties, including compressive strength, flexural strength, and splitting strength, but no consistent conclusions have been made.

As shown in Table 3, some researchers have observed that the use of SAP in the concrete reduced the mechanical properties of concrete, which is attributed to the voids vacated by the drying SAPs [88,89]. Additionally, this reduction in mechanical properties is due to the fact that SAPs require additional water for workability, leading to an increase in capillary pores in the hardened matrix. However, results from other researchers revealed that the mechanical properties of concrete were enhanced by the addition of SAP due to enhanced hydration [20,33,90,91,92,93,94]. It can offset the negative impact of SAP on mechanical properties by filling the voids formed after the release of water from the SAP. It is notable that the mechanical properties of concrete mixture modified with SAP are also influenced by the curing method [94,95,96,97,98,99,100]. Effective curing ensures adequate hydration and minimizes shrinkage, ultimately improving the mechanical performance of concrete. On the other hand, insufficient curing can worsen the negative effects of SAPs by creating voids in the concrete after water release, weakening the overall structure. Factors such as variations in temperature, humidity, and curing duration can significantly impact the final properties of SAP-modified concrete. To achieve the best results, it is important to find the right balance in SAP dosage, mix proportions, and curing conditions, as these elements collectively determine the strength and durability of the concrete.

It should be noted that achieving a consistent w/cm In a mixture may be challenging when an SAP is withdrawing water from the paste while in a fresh state. This may be a cause for the lack of consistent findings in the literature.

In conclusion, the use of SAP in concrete may lead to a reduction in mechanical properties at early ages, particularly if curing is inadequate. However, incorporating fibers or other additives can enhance performance, resulting in concrete modified with SAP being more durable over the long term.

### 4.4. Shrinkage

Mitigating shrinkage is one of the primary benefits of internal curing in concrete mixtures. Volumetric changes in cement paste may be caused by different kinds of shrinkage: chemical/autogenous, plastic, and drying shrinkage.

#### 4.4.1. Chemical/Autogenous Shrinkage

**Figure 4:**
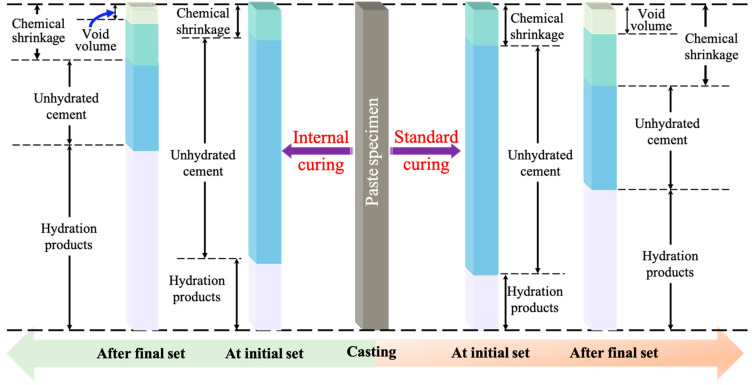
Chemical shrinkage of cement paste with (**left**) and without (**right**) SAP.

Chemical shrinkage is a result of the chemical reactions between cementitious materials and water resulting in products with reduced volume, which poses challenges to concrete strength and service life. The amount that can be measured is known as the autogenous shrinkage (as shown in Figure 4). Figure 4 shows the role of SAP in reducing chemical shrinkage in cement paste. It offers a comprehensive visualization of how SAPs can improve concrete durability by mitigating shrinkage and reducing potential cracking.

The SAP’s ability to mitigate autogenous shrinkage has been a major focus of research, especially for high performance cement-based mixtures with relatively low w/cm. Autogenous shrinkage, resulting from internal chemical reactions, can cause microcracking and reduced structural strength. It has been effectively addressed with the addition of SAP as an IC material. Researchers have demonstrated that the use of SAPs offsets the internal relative humidity and mitigates the corresponding autogenous shrinkage significantly, offering a promise for long-term durability. For instance, a study from thirteen international research groups was performed about the effect of two commercial SAPs and additional water on the autogenous shrinkage of concrete mixtures with the same composition but from local materials, the results from all groups showed that the autogenous shrinkage was successfully reduced, especially at early ages [103]. Considering early-age expansion and internal curing water, a model has been proposed to estimate SAP-cured concrete’s autogenous shrinkage [104]. As hydrated products such as ettringite and portlandite form, the growth of cement paste is believed to increase crystallization pressure resulting in the early-age expansion during hydration [105]. When embedded in concrete matrixes, SAPs act as reservoirs for internal curing water, absorbing and storing huge amounts of moisture. In the early stages of concrete formation, autogenous shrinkage is countered as cement hydrates, slowly releasing the stored water. As a result of maintaining higher relative humidity inside the concrete, SAPs prevent shrinkage and cracks, enhancing the structure’s durability and performance. With their dual function of absorbing water and releasing it, SAPs offer an effective way to deal with early-age drying and shrinkage in a range of concrete mixtures, including high-performance concrete and those incorporating cementitious additives [70,105]. In the context of contemporary green and sustainable development practices, mineral admixtures and supplementary cementitious materials (SCMs), such as fly ash and ground granulated blast furnace slag, are commonly integrated into concrete mixtures. Consequently, numerous studies have explored the potential impact of SAPs on shrinkage reduction in these composite materials [4,106,107,108]. Their results also proved that SAPs had the same efficiency in it.

In conclusion, SAPs are a multifaceted solution to concrete shrinkage, characterized by continuous improvements in compositions, dosages, and applications. Modern construction practices face a variety of challenges related to concrete shrinkage, and SAPs provide a promising solution. From fundamental understanding to real-world implementation, SAPs have proven to be a transformative technology, offering sustainable solutions designed to make concrete structures stronger and more durable. SAPs may have the potential to shape the future of concrete construction through continued synergy between research, industry applications, and material innovation.

#### 4.4.2. Plastic Shrinkage

Plastic shrinkage is developed in the concrete plastic stage caused by the capillary stresses due to water loss in fresh concrete. It occurs when the surface evaporation rate exceeds the rate at which the bleeding water reaches the surface of the concrete. The increase in capillary pressure and plastic shrinkage rate will yield tensile stress in the surface layers with reduced moisture, while restraint is provided by the non-contracting inner layers [109], resulting in short, shallow cracks that may encourage the ingress of aggressive solutions [110].

The risk of plastic shrinkage cracking is influenced by environmental humidity, temperature, winds, and surface area. Research has shown that the incorporation of SAP can reduce plastic shrinkage cracks by replacing water lost at the surface with evaporation [87,111,112,113,114].

#### 4.4.3. Drying Shrinkage

**Figure 5:**
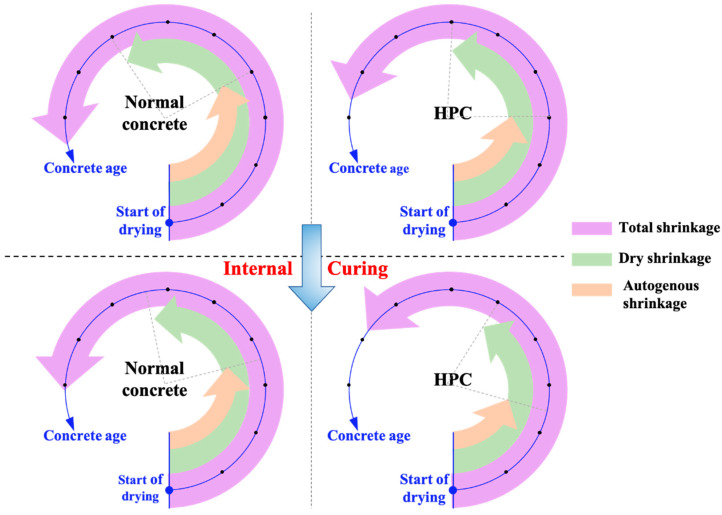
Drying shrinkage and autogenous with the concrete age in conventional concrete and high-performance concrete after internal curing.

Drying shrinkage is caused by water loss from hardened concrete. In terms of magnitude, it contributes most to the total shrinkage of a system [114,115] (as shown in Figure 5). In Figure 5, the reduction in drying and autogenous shrinkage over time is shown for conventional concrete and HPC when SAP is used. It emphasizes the positive effect of SAP on shrinkage mitigation, particularly in concrete mixtures with low water-to-cement ratios, which are more susceptible to shrinkage.

The drying shrinkage rate and the risk of cracking will decrease with internal curing. However, the efficiency of SAP on the drying shrinkage rate depends on the water-to-binders ratio used, the content and type of SAP added, the external environment, and the size of the specimen [70,91,96].

### 4.5. Permeability

The porosity, pore size, and pore connectivity will control the permeability of concrete [24,116]. The permeability is responsible for the entry of harmful substances like chlorides and carbon dioxide. The permeability of concrete modified with SAP should be reduced due to enhanced hydration. However, there are pores left by the SAP. It has been reported that SAP may swell and fill cracks if water penetrates the system [117,118]. For example, Snoeck et al. [119] used neutron radiography to observe healed cracks to show that SAP decreased the permeability of healed cracks up to 100 μm-wide.

Several papers have reported the influence of SAP on the permeability of concrete as shown in Table 4, including water permeability, oxygen permeability, capillary suction, chloride migration coefficient, and water absorption. The results show that incorporating SAPs can significantly decrease the permeability of concrete, thus improving its resistance to harmful elements like chlorides and carbon dioxide. The improved concrete properties resulting from the addition of SAP were attributed to its ability to improve the pore size distribution, facilitate the particle packing density, and enhance the interparticle bonding, leading to a lower porosity, and better strength and durability. The size of the voids created by SAPs is critical for the structural integrity of concrete. Smaller, evenly distributed voids can enhance overall toughness and resistance to cracking by ensuring a more uniform stress distribution under load. Conversely, larger voids can act as stress concentrators, potentially leading to crack propagation and failure under tensile or flexural loads. Additionally, the size and distribution of these voids directly impact the permeability of concrete. A concrete matrix with optimized void characteristics can minimize water ingress, improving resistance to environmental factors such as freeze–thaw cycles, chemical attacks, and carbonation [120].

The relationship between SAPs and permeability can be unraveled by understanding how SAPs influence permeability. One fundamental mechanism by which SAPs reduce concrete permeability is attributed to their impact on the microstructure. As their controlled moisture release helps prevent the formation of macro- and microcracks during the early stages of hydration, resulting in denser and more refined concrete with reduced pore sizes and improved particle packing [126,127], while the main formed hydration product at later ages is calcium carbonate crystallization that can range from macro-pore to micropore or heal the internal cracks [128,129]. The finer pore network acts as a barrier to fluid flow, lowering the permeability of the concrete. It is also pertinent to note that SAPs contribute to physical pore-blocking or crack-sealed, reducing permeability. Since SAPs absorb water, they undergo significant swelling, which leads to a significant blockage of capillaries and pores within the concrete as a result of swelling [130]. Due to this physical obstruction, water is impeded from moving freely through the interconnected pathways, resulting in reduced permeability [131]. In the event of an internal SAP, the cementitious material will crack, exposing the SAP particles. These swelling SAP particles will block a crack when fluid is introduced. It is noted that the SAP particles used are not too small for the expected crack widths as they may be washed out [132]. As a result of both internal curing and pore-blocking, the concrete is not only able to address shrinkage-related concerns, but it is also able to enhance its durability and imperviousness. These mechanisms have several advantages, including the ability to alleviate drying shrinkage and enhance concrete structures’ overall performance.

Moreover, the overall effectiveness of SAPs in modifying the microstructure is significantly influenced by environmental conditions. For example, in high humidity, SAPs may not effectively release water, resulting in less noticeable changes in microstructure. On the other hand, in low-humidity environments, SAPs may release water too rapidly, leading to increased capillary porosity and decreased strength [6,133]. Therefore, it is essential to understand the interaction between SAPs and environmental factors to optimize concrete performance.

In summary, the efficiency of SAP in decreasing the permeability of concrete is multifaceted. The outcome of experiments can vary due to different conditions, such as curing environments, concrete age, and SAP particle size. Furthermore, the level of permeability reduction depends on the extent to which the concrete matrix has internally cured, which in turn depends on the availability of water. Therefore, it is crucial for further research to customize SAP applications based on specific project requirements, such as water content, curing conditions, and SAP particle size in order to achieve the desired reduction in concrete permeability. Additionally, combining with other green cement-based materials like slag or fly ash [134,135], or expansive agents/crystalline admixture [136,137,138], or nanomaterials [40,139], or calcium carbonate precipitating bacteria [140,141,142], can further improve its efficiency on concrete properties.

### 4.6. Air Void Systems

The presence of a stable, adequate air void system is required for concrete exposed to freeze–thaw cycles. Chemical air-entraining agents (AEAs) are used to stabilize small air voids in a concrete mixture that act as pressure relief zones for water that moves and expands as it freezes [143]. However, air content created by AEAs is variable with the changes in temperature, haul time, and vibration of the fresh mixture.

SAP has the potential to act as a physical air void after desorption [33,144]. Caveats are that the spacing between voids may be higher than desired and that voids may become filled with hydration products [74,145]. SAP with an appropriate particle size has contributed to the air-void system [146,147]. Moreover, Mechtcherine et al. [148] compared the effect of traditional AEA and SAP on the air void system of concrete. the results revealed that SAP could create a more stable air void system in concrete compared to AEA, whereas it reduced the adverse effect of additional water on the mechanical performances of concrete compared with traditional AEA.

## 5. Specifications

Methods have been recommended by the American Concrete Institute (ACI) to mitigate early-age shrinkage cracking. These can be divided into three categories [149]. From the perspective of structures, the tensile strength of hardened concrete can be enhanced by adding reinforcing steel or fibers. From the perspective of materials, the consumption of water can be compensated by some internal curing using lightweight aggregates (LWA) or superabsorbent polymers (SAP). From the point of view of construction, external curing methods can be adopted to minimize the moisture loss of concrete such as water spraying, coverings, and membrane-forming curing compounds. However, this method often fails to provide sufficient moisture to the deeper concrete layers, leading to incomplete hydration and potential surface cracks.

To overcome the limitations of external curing, alternative methods are often employed. For instance, IC in cement-based composites is regarded as an effective method to improve the performance of mixtures with a low water-cementitious material ratio (w/cm), which is particularly significant for the shrinkage properties of concrete [150,151,152,153]. As an IC material, LWA is divided into lightweight fine aggregate (LWFA) and lightweight coarse aggregate (LWCA). However, LWFA is preferable to LWCA due to its uniform distribution in the matrix, relatively lower cost, and existing standards such as ASTM C1761 [154]. The dosage, pre-conditioning, mixing procedure, and other requirements are discussed in the specification [155,156,157,158,159]. It is also necessary to measure its properties such as total, absorbed, and surface moisture prior to adding it to the mixture. Technical standards like [159,160] can be used to complete these tests. Moreover, the amount of LWFA needed as an internally cured mixture in mix design is determined with a tool [159,161]. In summary, research, design, guidelines, and implementation of LWFA as IC have proven to be feasible and effective.

While LWFA has demonstrated its feasibility and effectiveness, it has a finite capacity to absorb and release water, and its effectiveness may diminish over time. Even the addition of LWFA may lead to an overall increase in the cost of concrete, potentially impacting its economic feasibility for specific applications. SAP is promising for use as an IC material, but it has not found common use in the USA. It is crucial to explore ways to align SAP applications with existing standards and practices in order to advance its utilization in the broader context of concrete technology. SAP can be included in the group of concrete admixtures according to EN 206, since its dosage is 0.3–0.6% of the binder mass [161,162]. Introducing SAP into the concrete mixture affects the absorption of a portion of the mixing water, dramatically exceeding the mass and volume of the added SAP. Therefore, SAP dosage should be based on the absorption rates of the product. A test method such as the “teabag method” is proposed that determines the free swell capacity of SAP powders in saline solution or other aqueous solution [159,163]. The detailed testing procedure for the absorption of SAP using teabags is described in [159]. This method also refers to the paste volume including SAP which shall be not more than 26.5% of the concrete design volume, and the maximum w/cm ratio (0.42) in concrete mixture design during construction.

Some reports and related documents involved the properties of SAP and other aspects such as its influences on the performances of concrete, potential applications, and case studies [148,164,165,166]. For example, a State-of-the-Art Report of TC 225-SAP published in 2012 summarized its scientific knowledge and potential application in the concrete mixture system, which focused on the improvement of the autogenous shrinkage and the freeze–thaw resistance of concrete [166]. Subsequently, TC 225-RSC started in 2014 primarily facilitated the incorporation of SAP in construction practice by preparing technical recommendations for its use in concrete [164]. However, the use of SAP at large scales is limited by challenges such as achieving uniform distribution and the absence of standardized implementation guidelines. Continued research is necessary to optimize SAP usage for widespread applications.

To ensure consistency in the absence of established standards, a comprehensive framework is recommended for researchers and engineers. First, detailed documentation of procedures, including material specifications, mixing processes, and testing methods, should be maintained to enable replication across projects. Establishing performance indicators like shrinkage reduction, workability, rheology, and durability will create a clear basis for comparison and evaluation. Lastly, a collaborative approach that involves sharing methods, results, and peer-reviewed feedback will foster best practices. These guidelines will lay a foundation for consistent practices, ultimately supporting the development of formal standards.

## 6. Case Studies on the Use of SAP in Construction Projects

**Figure 6:**
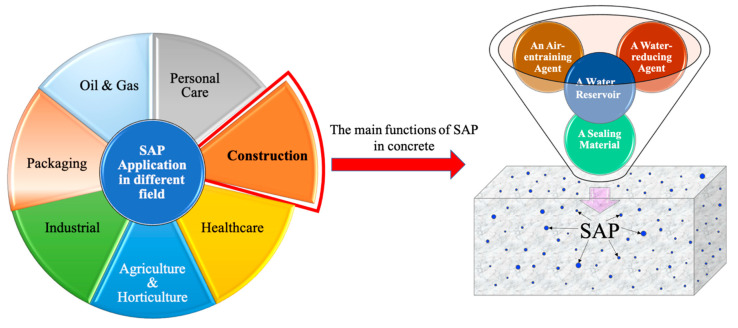
Different applications of SAP and its functions in concrete.

As mentioned in the previous sections, SAPs have been utilized in various fields such as agriculture, wastewater treatment, hygiene products, pharmaceuticals, and food packaging, among others, as shown in Figure 6, owing to their high water absorption capacity, and controlled release kinetics. Moreover, they can be compatible with cementitious materials, making them promising additives for a variety of concrete applications, including road construction and infrastructure projects to sustainable building materials and precast concrete manufacturing. Following are some structures modified with SAP in order to achieve improved performance of the mixture.

### 6.1. Reduced Shrinkage Cracking

(a) The 2006 FIFA World Cup Stadium in Kaiserslautern, Germany

The first construction project in the world that used SAP in a concrete structure was a pavilion built for the 2006 FIFA World Cup in Kaiserslautern, Germany [100]. The pavilion was designed as a filigreed, thin-walled high-performance concrete structure with thin columns and no conventional reinforcement. Control performance parameters included reduced autogenous shrinkage, high durability, enhanced ductility, and self-desiccation were developed [100]. However, its applicability is limited to structures that require traditional reinforcement.

(b) China

Some examples using concrete modified with SAP as an internal curing agent have been constructed in large-scale structures in China. A second double-lane of the Lanzhou-Urumgi railway was built using 1,050,000 yd^3^ of bed slab concrete designed with w/cm of 0.37 in a windy and arid environment [167]. SAP was incorporated along with shrinkage-reducing admixtures to reduce the risk of moisture loss due to evaporation, thus reducing the risk of cracking due to plastic shrinkage [71,168].

A 65′ high × 235′ long × 3′ wide shear wall was built containing a novel kind of SAP [71]. Field tests showed that no cracks formed in the SAP section for up to seven days, while cracks were observed in the concrete containing only an expansion agent.

SAP used as a type of chemical admixture was also applied in the construction of the China Zun tower [169,170]. The results showed that when SAP content was 0.56 m%, the shrinkage rate decreased by 46%, and the later compressive strength was not affected.

However, it is important to consider the potential cost increases associated with the integration of SAP in practical applications. Additionally, there is the challenge of evenly distributing SAP in large-scale projects and ensuring consistent performance throughout various sections of the structure.

### 6.2. Freeze–Thaw Resistance of Concrete

The use of SAP can increase the freeze–thaw resistance of a mixture. It is attributed to the capabilities of SAP to absorb and retain water, which ensures concrete does not dry out and crack during freeze–thaw cycles. At the same time, after the water release from swollen SAP particles timely, distinctive macro pores with corresponding shapes are created. These pores fall within the size range that proves effective in improving freezing-and-thawing resistance, whether in the presence or absence of deicing salts [146,165,171,172]. Additionally, SAP can reduce concrete permeability significantly. By reducing the microcracks, water encounters more resistance when penetrating deeper into concrete during freeze–thaw cycles [147,173,174]. In this way, repeated freezing and thawing cycles serve as a protective barrier against further damage. Furthermore, SAP enhances concrete’s overall durability and resistance to freeze–thaw damage by refining its microstructure within cement paste. The improvement of the frost resistance of highly ductile short-fiber reinforced concrete (SHCC) with SAP was investigated by researchers at Technische Universität Dresden. In the summer of 2011, the practical worldwide application of SAP was used as an additive to increase the freeze–thaw resistance of SHCC to repair the upper water reservoir of the pumping hydraulic power station Hohenwarte II in Thüringen, Germany [168]. The risk of freezing damage to reservoir walls was high due to its saturation and variation of water level. SAP was utilized as an additive to increase the frost resistance of the repair layer [168,175]. However, further research is required to optimize the size and distribution of SAP particles in the concrete matrix to improve freeze–thaw resistance. Moreover, studies should investigate how different SAP compositions perform under various environmental conditions, such as exposure to de-icing salts, as these factors may affect the long-term durability of the material in engineering applications.

### 6.3. Improved Rheology

Based on a 1991 patent application from Snashall [176], the subject of using SAP was to increase viscosity and decrease rebound of shotcrete, they recommended premix SAP with the aggregate and 10–15% of the aggregate weight of water, followed by stand-in the mixer for 10 min, and finally, the cement and the rest of the mix water were incorporated. The addition ways of SAP had two functions according to the patent application from Jensen and Hansen [177], one is to add dry SAP to the nozzle to reduce the viscosity of a wet mixture, which requires SAP to absorb water quickly to achieve the desired viscosity before the shotcrete hits the wall; the other is to incorporate the presoaked or partially presoaked SAP into the concrete for internal curing purposes. For example, SAP-modified concrete was applied to shotcrete the wall panels in Lyngby, Denmark [178]. In this case, the presence of 0.4% SAP in shotcrete with a water-to-cement of 0.4 acted as a rheology modifier, because no other set-accelerating admixture was adopted in this construction project. However, it is critical to optimize the timing and method of adding SAP during mixing based on specific SAP properties, which can help ensure better consistency and avoid negative impacts on strength and durability.

### 6.4. Self-Sealing

When SAP comes into contact with water, it absorbs water and expands to form a flow barrier. Based on this, the sealing composite materials were made by mixing modified SAP with rubber as described by Tsubakimoto et al. [179]. These composite materials have been used to seal joints of various building materials. They may be used to plug any gaps left by settlement during or after construction like mortar [180]. According to reports [181], this composite material was employed in the construction of the Channel Tunnel between France and England. While SAP facilitates self-sealing by swelling upon moisture contact, filling micro-cracks, and blocking water and harmful agents, further research is required to evaluate SAP’s long-term effectiveness across diverse environmental conditions and its potential impact on large-scale structures.

In summary, these applications demonstrate SAP’s role in improving concrete performance across various implementations while also identifying areas that require further research and optimization to fully realize its benefits in real-world projects.

## 7. Conclusions and Prospects

IC has gained attention as a method to enhance concrete performance by providing additional water during the hydration process. Superabsorbent polymers (SAPs), in particular, offer a promising solution due to their ability to absorb and retain significant amounts of water and gradually release it as needed. This helps mitigate common issues like shrinkage and cracking, improving the durability and workability of concrete.

SAPs have been widely studied and applied in HPC, where they reduce autogenous and drying shrinkage, enhance hydration, and improve long-term durability. However, the effectiveness of SAPs is closely tied to their physical and chemical properties, such as absorption rate, desorption behavior, particle size, and crosslinking density. SAPs must be carefully selected based on the specific requirements of the concrete mix to ensure optimal performance.

Despite their benefits, challenges remain in the application of SAPs in concrete mixtures. Achieving a uniform distribution of SAP particles is difficult, especially in large-scale applications. Additionally, the lack of standardized guidelines for SAP usage complicates their implementation across different construction projects. Further research is needed to better understand the impact of SAPs on transport properties, frost durability, and the workability of concrete under various conditions.

Case studies demonstrate the successful use of SAPs in reducing shrinkage and enhancing durability in large-scale infrastructure projects, particularly in harsh environmental conditions. However, the current knowledge gap in the standardization of SAP use and its effect on normal-strength concrete calls for further investigation.

In conclusion, SAPs hold great potential for improving concrete performance, particularly in reducing shrinkage and enhancing durability. Future research should focus on optimizing SAP selection and dosage, as well as developing standardized methods for their integration into concrete mixtures. These advancements will contribute to the broader adoption of SAPs in sustainable construction practices, extending the service life of concrete structures and reducing maintenance costs.

## Figures and Tables

**Table 1:** Mixing procedure of SAP-modified concrete mixtures.

References	Mixing Procedure	References	Mixing Procedure
[52]	(1) mixing aggregates and cementitious materials for 60 s; (2) adding water and superplasticizer and mixing for 5 min; (3) adding pre-saturated sap and mixing for 2 min.	[67]	(1) mixing cement powder, oven-dried-aggregates, and dry SAP for 30 s; (2) adding the first half of water and mixing for 120 s; (3) adding the second half of water together with the SP and mixing for 5 min.
[68]	(1) prewetting the SAP; (2) mixing the prewetted SAP and other dry materials for 60 s; (3) adding water and mixing for 60 s.	[69]	(1) mixing fine aggregate, coarse aggregate, cement, and fly ash for 30 s; (2) adding Water reducing agent and concrete mixing water and mixing for 100 s; (3) adding non-absorbed or pre-absorbed sap and mixing for 60 s.
[70]	(1) mixing cement, sand, and aggregate for 1 min; (2) adding water together with the pre-soaked SAP to the dry mixture and mixing for 3 min.	[71]	(1) mixing gravel, sand, cement, GGBS, and fly ash for 15 s; (2) adding the water containing superplasticizer and the diluted ICA (with 50% of mixing water) and mixing for 5 min.
[72]	(1) mixing cement, sand, and aggregate for 1 min; (2) adding water together with the pre-soaked SAP to the dry mixture and mixing for 3 min.	[73]	(1) mixing cement, fine aggregate, superplasticizer, and saturated sap for 1 min; (2) adding water and mixing for 40 s and then adding fibers
[74]	(1) mixing SAP and all dry cementitious materials for 20 s; (2) adding approximately 90% of water during the period of 30 s; (3) wet-mixing for 60 s; (4) adding SP, the rest of water and mixing for 90 s; (5) adding fiber during the period of 45 s; (6) mixing for 120 s.	[75]	(1) mixing dry materials for 1 min (including SAPs); (2) adding water and superplasticizer and mixing for additional 2 min; (3) adding the additional entrained water and mixing for additional 2 min.

**Table 2:** Influences of SAP-modified concrete mixtures.

Concrete Types	Adding Methods of SAP	Influences on the Concrete Properties
SCLWC [52]	pre-soaked	(a) improves passing ability and decreases the static stability. (b) reduces the compressive strength. (c) induces a negative effect on the electrical resistivity and water absorption. (d) reduces the total shrinkage.
HPC [68]	prewetted	(a) obviously delays internal relative humidity (IRH) decline. (b) mitigates autogenous shrinkage (AS) at an early age. (c) decreases the compressive strength.
High-strength concrete [70]	pre-soaked	(a) firmly alleviates the early-age shrinkage related to moisture loss. (b) postpones the drop in internal humidity. (c) slightly reduces the compressive strength.
HPC [72]	pre-soaked	(a) increases the total porosity of the hardened cement paste. (b) increases the gel pores and small capillary pores. (c) increases the chloride diffusion coefficient.
UHPC [74]	dry	(a) reduces the internal relative humidity decrease and the autogenous shrinkage. (b) decreases the compressive strength, flexural strength, and elastic modulus. (c) delays the hydration time of the mixtures.
HPC [67]	dry	(a) no change in the compressive strength over 28 days. (b) reduces autogenous shrinkage and drying shrinkage. (c) significantly decreases the tensile creep of SAP modified concrete.
Normal-strength concrete [69]	non-absorbed/pre-absorbed	(a) increases compressive strength. (b) reduces the shrinkage. (c) improves the carbonation resistance and chloride penetration resistance. (d) effectively improves the microstructure and pore structure.
Normal-strength concrete [71]	gel-type internal curing agent (ICA)	(a) reduces the cracking area of concrete. (b) delays the rates of decline in the IRH. (c) mitigates early-age cracking.
Cellular concrete [73]	millimeter-size spherical saturated SAP	(a) decreases the compressive strength. (b) increases the average dynamic factor. (c) decreases the dynamic increase factor.
Normal-strength concrete [75]	dry	(a) no significant influence on the total shrinkage or the compressive strength. (b) increases the number of bigger pores. (c) decreases the shrinkage strain of concrete

**Table 3:** Effect of SAP on the mechanical properties of concrete at different ages.

Concrete Types	w/c	Changes in Compressive Strength (%)			Content of SAP (wt.%)
		7d	28d	90d	
UHPC [74]	0.15	−13.5	−9.3	−13.5	0.206
		−29.3	−19	−21.6	0.313
HPC [76]	0.25	−10.2	−0.9	23.9	0.18
		−16.0	4.7	4.8	0.2
HPC [69]	0.38	−2.1	−4.2	−4.1	0.1
		−4.2	−5.1	−3.3	0.2
		−6.3	−5.9	−4.9	0.3
Normal concrete [101]	0.45	39.9	3.6	⏤	0.1
		45.9	5.2	⏤	0.2
		49.7	9.2	⏤	0.3
		37.6	6.0	⏤	0.4
Normal concrete [94,102]	0.5	19.5	1.2	⏤	0.2
	0.4	−4.4	−9.8	⏤	0.2
	0.35	−1.7	−4.3	⏤	0.2
Normal concrete [94]	0.35	4.0	2.1	⏤	0.1
		39.9	3.6	⏤	0.2
		45.9	5.2	⏤	0.3
		49.7	9.2	⏤	0.4
UHPC [31]	0.3	−9.9	−5	−4.9	0.2
		−16.5	−11.7	−11.5	0.4
		−31.9	−15.8	−13.9	0.6
HPC [88]	0.3	−4.4	−6.7	⏤	0.57
		−9.2	−12.8	⏤	0.86
		−31.3	−26.1	⏤	1.14

Note: “⏤” symbol means there were no test results at corresponding ages collected by the authors.

**Table 4:** Effect of SAP on the permeability of concrete.

Evaluation Indexes	Changes Compared to the Reference Without SAP	SAP Contents	References
Water permeability	Same or decrease	0.33%, 0.58% and 0.25%	[121,122]
Oxygen permeability	Same or decrease		
Capillary suction	Decrease		
Chloride migration coefficient	Decrease	0.05%, 0.1%, 0.2% and 0.3%	[78]
Chloride migration coefficient	Decrease	0.3%	[123]
Carbonation depth Chloride migration coefficient	Decrease	0.1%, 0.2% and 0.3%	[69]
Water absorption	Decrease	0.3%, 0.6% and 0.9%	[124]
Chloride ion diffusion coefficient	Decrease		
Carbonation depth	Decrease		
Gaseous and fluid permeability	Decrease	---	[125]

## Data Availability

No new data were created or analyzed in this study.

## References

[1] De Meyst L., Mannekens E., Van Tittelboom K., De Belie N. (2021). The Influence of Superabsorbent Polymers (SAPs) on Autogenous Shrinkage in Cement Paste, Mortar and Concrete. Constr. Build. Mater..

[2] Wyrzykowski M., Assmann A., Hesse C., Lura P. (2020). Microstructure Development and Autogenous Shrinkage of Mortars with C-S-H Seeding and Internal Curing. Cem. Concr. Res..

[3] Guan X., Zhang J., Zhao S. (2024). Design, Synthesis and Characterization of a Starch-Based Superabsorbent Polymer and Its Impact on Autogenous Shrinkage of Cement Paste. Constr. Build. Mater..

[4] Snoeck D., Jensen O.M., De Belie N. (2015). The Influence of Superabsorbent Polymers on the Autogenous Shrinkage Properties of Cement Pastes with Supplementary Cementitious Materials. Cem. Concr. Res..

[5] Hilloulin B., Tran V.Q. (2022). Using Machine Learning Techniques for Predicting Autogenous Shrinkage of Concrete Incorporating Superabsorbent Polymers and Supplementary Cementitious Materials. J. Build. Eng..

[6] Hong G., Kim J., Song C., Yeon J.H., Choi S. (2024). Effect of Internal Curing by Superabsorbent Polymers on the Densification and Microstructural Development of Cementitious Materials Exposed to Different Environmental Conditions. Constr. Build. Mater..

[7] Álvarez M., Ferrández D., Fernández C.M., Atanes-Sánchez E. (2024). Super Absorbent Polymers (SAP) in Building Materials: Application Opportunities through Physico-Chemical and Mechanical Analysis. Constr. Build. Mater..

[8] Tutkun B., Yazıcı H. (2023). Effect of Absorption Determining Methods of Superabsorbent Polymers in Cementitious Environments on the Fresh Properties. Mater. Today Proc..

[9] Gu Y., Mohseni E., Farzadnia N., Khayat K.H. (2024). An Overview of the Effect of SAP and LWS as Internal Curing Agents on Microstructure and Durability of Cement-Based Materials. J. Build. Eng..

[10] Xu F., Lin X., Zhou A. (2021). Performance of Internal Curing Materials in High-Performance Concrete: A Review. Constr. Build. Mater..

[11] Kelly S.L., Krafcik M.J., Erk K.A. (2018). Synthesis and Characterization of Superabsorbent Polymer Hydrogels Used as Internal Curing Agents: Impact of Particle Shape on Mortar Compressive Strength. Proceedings of the International Congress on Polymers in Concrete (ICPIC 2018).

[12] Ma X., Wen G. (2020). Development History and Synthesis of Super-Absorbent Polymers: A Review. J. Polym. Res..

[13] Santos R.V.A., Costa G.M.N., Pontes K.V. (2019). Development of Tailor-Made Superabsorbent Polymers: Review of Key Aspects from Raw Material to Kinetic Model. J. Polym. Environ..

[14] Miyajima T., Matsubara Y., Komatsu H., Miyamoto M., Suzuki K. (2019). Development of a Superabsorbent Polymer Using Iodine Transfer Polymerization. Polym. J..

[15] Mignon A., De Belie N., Dubruel P., Van Vlierberghe S. (2019). Superabsorbent Polymers: A Review on the Characteristics and Applications of Synthetic, Polysaccharide-Based, Semi-Synthetic and ‘Smart’ Derivatives. Eur. Polym. J..

[16] Venkatachalam D., Kaliappa S. (2023). Superabsorbent Polymers: A State-of-Art Review on Their Classification, Synthesis, Physicochemical Properties, and Applications. Rev. Chem. Eng..

[17] Saha A., Sekharan S., Manna U. (2020). Superabsorbent Hydrogel (SAH) as a Soil Amendment for Drought Management: A Review. Soil. Tillage Res..

[18] Bora A., Karak N. (2022). Starch and Itaconic Acid-Based Superabsorbent Hydrogels for Agricultural Application. Eur. Polym. J..

[19] Zhao C., Zhang L., Zhang Q., Wang J., Wang S., Zhang M., Liu Z. (2022). The Effects of Bio-Based Superabsorbent Polymers on the Water/Nutrient Retention Characteristics and Agricultural Productivity of a Saline Soil from the Yellow River Basin, China. Agric. Water Manag..

[20] Al Saffar D.M., Al Saad A.J.K., Tayeh B.A. (2019). Effect of Internal Curing on Behavior of High Performance Concrete: An Overview. Case Stud. Constr. Mater..

[21] Sujitha V.S., Ramesh B., Xavier J.R. (2023). Effect of Superabsorbent Polymer Hydrogels in the Advancement of Cementitious Materials—A Review. J. Polym. Environ..

[22] Xie Z., Yao H., Yuan Q., Zhong F. (2023). The Roles of Water-Soluble Polymers in Cement-Based Materials: A Systematic Review. J. Build. Eng..

[23] Danish A., Mosaberpanah M.A., Salim M.U. (2020). Past and Present Techniques of Self-Healing in Cementitious Materials: A Critical Review on Efficiency of Implemented Treatments. J. Mater. Res. Technol..

[24] Liu R., Xiao H., Liu J., Guo S., Pei Y. (2019). Improving the Microstructure of ITZ and Reducing the Permeability of Concrete with Various Water/Cement Ratios Using Nano-Silica. J. Mater. Sci..

[25] Shi P., Falliano D., Vecchio F., Marano G.C. (2024). Investigation on the Compressive Strength and Durability Properties of Alkali-Activated Slag Mortar: Effect of Superabsorbent Polymer Dosage and Water Content. Dev. Built Environ..

[26] Sujitha V.S., Ramesh B., Xavier J.R. (2023). Influence of Nano Alumina Reinforced Superabsorbent Polymer on Mechanical, Durability, Microstructural and Rheological Properties of Cementitious Materials. J. Build. Eng..

[27] Hung C.C., Atmajayanti A.T., Meiji V.C.D., Mo K.H., Yoo D.Y. (2024). Performance of High-Strength Green Strain-Hardening Cementitious Composites Incorporating CSA/CAC Cements and GGBS. Structures.

[28] Paul A., John E. (2023). Study on the Optimisation of Cement and Binder Content to Develop a Sustainable High-Performance Concrete. Mater. Today Proc..

[29] Yang L., Shi C., Liu J., Wu Z. (2021). Factors Affecting the Effectiveness of Internal Curing: A Review. Constr. Build. Mater..

[30] Jensen O.M., Hansen P.F., Lachowski E.E., Glasser F.P. (1999). Clinker Mineral Hydration at Reduced Relative Humidities. Cem. Concr. Res..

[31] Liu J., Farzadnia N., Khayat K.H., Shi C. (2021). Effects of SAP Characteristics on Internal Curing of UHPC Matrix. Constr. Build. Mater..

[32] Somers M.J., Alfaro J.F., Lewis G.M. (2021). Feasibility of Superabsorbent Polymer Recycling and Reuse in Disposable Absorbent Hygiene Products. J. Clean. Prod..

[33] Jensen O.M., Hansen P.F. (2001). Water-Entrained Cement-Based Materials: I. Principles and Theoretical Background. Cem. Concr. Res..

[34] English A.E., Tanaka T., Edelman E.R. (1998). Polymer and Solution Ion Shielding in Polyampholytic Hydrogels. Polymers.

[35] Schröfl C., Mechtcherine V., Gorges M. (2012). Relation between the Molecular Structure and the Efficiency of Superabsorbent Polymers (SAP) as Concrete Admixture to Mitigate Autogenous Shrinkage. Cem. Concr. Res..

[36] Mignon A., Graulus G.J., Snoeck D., Martins J., De Belie N., Dubruel P., Van Vlierberghe S. (2014). PH-Sensitive Superabsorbent Polymers: A Potential Candidate Material for Self-Healing Concrete. J. Mater. Sci..

[37] Zhu H., Wang Z., Xu J., Han Q. (2019). Microporous Structures and Compressive Strength of High-Performance Rubber Concrete with Internal Curing Agent. Constr. Build. Mater..

[38] Mönnig S. (2015). Superabsorbing Additions in Concrete: Applications, Modelling and Comparison of Different Internal Water Sources. Master’s Thesis.

[39] Yang J., Liu Y., Zeng J., Su Y., Wang F., He X. (2024). Performance Enhancement of Cement Mortar with Superabsorbent Polymer Composite Internally Embedded with Porous Ceramsite Sand. J. Build. Eng..

[40] Shen Y., Wang Z., Zhou Y., Du J., Lai J., Qian K., Ruan S., Bi Y., Qian X. (2024). The Influence of Nano-Silica Composite Superabsorbent Polymer on the Autogenous Shrinkage of Mortar. Constr. Build. Mater..

[41] Yang J., Wang F., Liu Z., Liu Y., Hu S. (2019). Early-State Water Migration Characteristics of Superabsorbent Polymers in Cement Pastes. Cem. Concr. Res..

[42] Chen S., Xiang Z., Yao N., Liu G., Hou C., Li Z. (2024). Effects of Superabsorbent Polymer on Mechanical Properties of Cemented Paste Backfill and Its Mechanism Evolution. Constr. Build. Mater..

[43] Dodangeh F., Nabipour H., Rohani S., Xu C. (2024). Applications, Challenges and Prospects of Superabsorbent Polymers Based on Cellulose Derived from Lignocellulosic Biomass. Bioresour. Technol..

[44] Ashkani M., Kabiri K., Salimi A., Bouhendi H., Omidian H. (2018). Hybrid Hydrogel Based on Pre-Gelatinized Starch Modified with Glycidyl-Crosslinked Microgel. Iran. Polym. J..

[45] Li X., Shu M., Li H., Gao X., Long S., Hu T., Wu C. (2018). Strong, Tough and Mechanically Self-Recoverable Poly(Vinyl Alcohol)/Alginate Dual-Physical Double-Network Hydrogels with Large Cross-Link Density Contrast. RSC Adv..

[46] Jeong D., Kim C., Kim Y., Jung S. (2020). Dual Crosslinked Carboxymethyl Cellulose/Polyacrylamide Interpenetrating Hydrogels with Highly Enhanced Mechanical Strength and Superabsorbent Properties. Eur. Polym. J..

[47] Mohammadbagheri Z., Rahmati A., Hoshyarmanesh P. (2021). Synthesis of a Novel Superabsorbent with Slow-Release Urea Fertilizer Using Modified Cellulose as a Grafting Agent and Flexible Copolymer. Int. J. Biol. Macromol..

[48] Ma S., Liu M., Chen Z. (2004). Preparation and Properties of a Salt-Resistant Superabsorbent Polymer. J. Appl. Polym. Sci..

[49] Esteves L.P. (2011). Superabsorbent Polymers: On Their Interaction with Water and Pore Fluid. Cem. Concr. Compos..

[50] Kazemian M., Shafei B. (2024). Investigation of Type, Size, and Dosage Effects of Superabsorbent Polymers on the Hydration Development of High-Performance Cementitious Materials. Constr. Build. Mater..

[51] Chen F., Bai S., Guan X., Qiao J., Gou H. (2024). Influence of Type and Particle Size of Superabsorbent Polymer on Early Water Distribution and Internal Curing Zone Properties of Cement Paste. Cem. Concr. Compos..

[52] Azarijafari H., Kazemian A., Rahimi M., Yahia A. (2016). Effects of Pre-Soaked Super Absorbent Polymers on Fresh and Hardened Properties of Self-Consolidating Lightweight Concrete. Constr. Build. Mater..

[53] Deng H., Liao G. (2018). Assessment of Influence of Self-Healing Behavior on Water Permeability and Mechanical Performance of ECC Incorporating Superabsorbent Polymer (SAP) Particles. Constr. Build. Mater..

[54] De Meyst L., Mannekens E., Araújo M., Snoeck D., Van Tittelboom K., Van Vlierberghe S., De Belie N. (2019). Parameter Study of Superabsorbent Polymers (SAPs) for Use in Durable Concrete Structures. Materials.

[55] Ma X., Yuan Q., Liu J., Shi C. (2019). Effect of Water Absorption of SAP on the Rheological Properties of Cement-Based Materials with Ultra-Low w/b Ratio. Constr. Build. Mater..

[56] Tangkokiat P., Thanapornpavornkul T., Muangkaew S., Siriwatwechakul W., Siramanont J., Snguanyat C. (2020). Characterization of Neutral Versus Anionic Superabsorbent Polymers (SAPs) in Ion-Rich Solutions for Their Use as Internal Curing Agents. RILEM Bookseries.

[57] Jensen O. Water Absorption of Superabsorbent Polymers in a Cementitious Environment. Proceedings of the International RILEM Conference on Advances in Construction Materials through Science and Engineering.

[58] Schröfl C., Erk K.A., Siriwatwechakul W., Wyrzykowski M., Snoeck D. (2022). Recent Progress in Superabsorbent Polymers for Concrete. Cem. Concr. Res..

[59] He R., Tan Y., Chen H., Wang Z., Zhang J., Fang J. (2020). Preparation and Properties of Novel Superabsorbent Polymer (SAP) Composites for Cementitious Materials Based on Modified Metakaolin. Constr. Build. Mater..

[60] Zhong P., Wyrzykowski M., Toropovs N., Li L., Liu J., Lura P. (2019). Internal Curing with Superabsorbent Polymers of Different Chemical Structures. Cem. Concr. Res..

[61] Zohuriaan-Mehr M.J. (2006). Super-Absorbents. Iran. Polym. Soc. Tehran.

[62] Jabbar Braihi A., Issa Salih S., Abbas Hashem F., Kareem Ahmed J., Author C. (2014). Proposed Cross-Linking Model for Carboxymethyl Cellulose/Starch Superabsorbent Polymer Blend. Int. J. Mater. Sci. Appl..

[63] Serafim A., Curti F., Olăret E., Nicolae C., Stancu I.C. (2021). Superabsorbent Polymers for Nanomaterials. Superabsorbent Polymers: Chemical Design, Processing and Applications.

[64] English A.E., Edelman E.R., Tanaka T. (2000). Polymer Hydrogel Phase Transitions. Experimental Methods in Polymer Science: Modern Methods in Polymer Research and Technology.

[65] Richter A., Paschew G., Klatt S., Lienig J., Arndt K.F., Adler H.J.P. (2008). Review on Hydrogel-Based PH Sensors and Microsensors. Sensors.

[66] Kang S.H., Hong S.G., Moon J. (2018). Importance of Monovalent Ions on Water Retention Capacity of Superabsorbent Polymer in Cement-Based Solutions. Cem. Concr. Compos..

[67] Assmann A., Reinhardt H.W. (2014). Tensile Creep and Shrinkage of SAP Modified Concrete. Cem. Concr. Res..

[68] Wang F., Zhou Y., Peng B., Liu Z., Hu S. (2009). Autogenous Shrinkage of Concrete with Super-Absorbent Polymer. Mater. J..

[69] Dang J., Zhao J., Du Z. (2017). Effect of Superabsorbent Polymer on the Properties of Concrete. Polymers.

[70] Kong X.M., Zhang Z.L., Lu Z.C. (2015). Effect of Pre-Soaked Superabsorbent Polymer on Shrinkage of High-Strength Concrete. Mater. Struct..

[71] Liu R., Sun Z., Asce A.M., Ding Q., Chen P., Chen K. (2017). Mitigation of Early-Age Cracking of Concrete Based on a New Gel-Type Superabsorbent Polymer. J. Mater. Civil. Eng..

[72] Xiangmin K. (2013). Effect of Super-Absorbent Polymer on Pore Structure of Hardened Cement Paste in High-Strength Concrete. J. Chin. Ceram. Soc..

[73] Deng Z., Cheng H., Wang Z., Zhu G., Zhong H. (2016). Compressive Behavior of the Cellular Concrete Utilizing Millimeter-Size Spherical Saturated SAP under High Strain-Rate Loading. Constr. Build. Mater..

[74] Justs J., Wyrzykowski M., Bajare D., Lura P. (2015). Internal Curing by Superabsorbent Polymers in Ultra-High Performance Concrete. Cem. Concr. Res..

[75] Tenório Filho J.R., Snoeck D., De Belie N. (2020). Mixing Protocols for Plant-Scale Production of Concrete with Superabsorbent Polymers. Struct. Concr..

[76] Savva P., Petrou M.F. (2018). Highly Absorptive Normal Weight Aggregates for Internal Curing of Concrete. Constr. Build. Mater..

[77] Kang S.H., Hong S.G., Moon J. (2017). Absorption Kinetics of Superabsorbent Polymers (SAP) in Various Cement-Based Solutions. Cem. Concr. Res..

[78] Hasholt M.T., Jensen O.M. (2015). Chloride Migration in Concrete with Superabsorbent Polymers. Cem. Concr. Compos..

[79] Beushausen H., Gillmer M. (2014). The Use of Superabsorbent Polymers to Reduce Cracking of Bonded Mortar Overlays. Cem. Concr. Compos..

[80] Lura P., Wyrzykowski M., Tang C., Lehmann E. (2014). Internal Curing with Lightweight Aggregate Produced from Biomass-Derived Waste. Cem. Concr. Res..

[81] Snoeck D., Schaubroeck D., Dubruel P., De Belie N. (2014). Effect of High Amounts of Superabsorbent Polymers and Additional Water on the Workability, Microstructure and Strength of Mortars with a Water-to-Cement Ratio of 0.50. Constr. Build. Mater..

[82] Liu J., Farzadnia N., Shi C., Ma X. (2019). Effects of Superabsorbent Polymer on Shrinkage Properties of Ultra-High Strength Concrete under Drying Condition. Constr. Build. Mater..

[83] Assmann A. (2013). Physical Properties of Concrete Modified with Superabsorbent Polymers. Master’s Thesis.

[84] Snoeck D., Schröfl C., Mechtcherine V. (2018). Recommendation of RILEM TC 260-RSC: Testing Sorption by Superabsorbent Polymers (SAP) Prior to Implementation in Cement-Based Materials. Mater. Struct..

[85] Schröfl C., Snoeck D., Mechtcherine V. (2017). A Review of Characterisation Methods for Superabsorbent Polymer (SAP) Samples to Be Used in Cement-Based Construction Materials: Report of the RILEM TC 260-RSC. Mater. Struct..

[86] Liu Y., Wei Y., Ma L., Wang L. (2022). Restrained Shrinkage Behavior of Internally-Cured UHPC Using Calcined Bauxite Aggregate in the Ring Test and UHPC-Concrete Composite Slab. Cem. Concr. Compos..

[87] Rostami R., Klemm A.J. Effect of Superabsorbent Polymers on Plastic Shrinkage Cracking and Properties of Fresh State Mortars Reinforced by Polymeric Fibres. Proceedings of the International Conference on Sustainable Materials, Systems and Structures (SMSS2019).

[88] Shen D., Liu C., Jiang J., Kang J., Li M. (2020). Influence of Super Absorbent Polymers on Early-Age Behavior and Tensile Creep of Internal Curing High Strength Concrete. Constr. Build. Mater..

[89] Lei X., Wang R., Jiang H., Xie F., Bao Y. (2020). Effect of Internal Curing with Superabsorbent Polymers on Bond Behavior of High-Strength Concrete. Adv. Mater. Sci. Eng..

[90] Ghourchian S., Wyrzykowski M., Lura P., Shekarchi M., Ahmadi B. (2013). An Investigation on the Use of Zeolite Aggregates for Internal Curing of Concrete. Constr. Build. Mater..

[91] Jensen O.M., Hansen P.F. (2002). Water-Entrained Cement-Based Materials: II. Experimental Observations. Cem. Concr. Res..

[92] Liu J., Shi C., Ma X., Khayat K.H., Zhang J., Wang D. (2017). An Overview on the Effect of Internal Curing on Shrinkage of High Performance Cement-Based Materials. Constr. Build. Mater..

[93] Memon R.P., Sam A.R.M., Awang A.Z., Tahir M.M., Mohamed A., Kassim K.A., Ismail A. (2020). Introducing Effective Microorganism as Self-Curing Agent in Self-Cured Concrete. IOP Conf. Ser. Mater. Sci. Eng..

[94] Piérard J., Pollet V., Cauberg N. Mitigating Autogenous Shrinkage in HPC by Internal Curing Using Super Absorbent Polymers. Proceedings of the International RILEM Conference on Volume Changes of Hardening Concrete: Testing and Mitigation.

[95] Craeye B., De Schutter G. (2009). Experimental Evaluation of Mitigation of Autogenous Shrinkage by Means of a Vertical Dilatometer for Concrete. Creep, Shrinkage and Durability Mechanics of Concrete and Concrete Structures, Proceedings of the 8th International Conference on Creep, Shrinkage and Durability Mechanics of Concrete and Concrete Structures, Ise-Shima, Japan, 30 September–2 October 2008.

[96] Igarashi S. (2006). Experimental Study on Prevention of Autogenous Deformation by Internal Curing Using Super-Absorbent Polymer Particles. International RILEM Conference on Volume Changes of Hardening Concrete: Testing and Mitigation.

[97] Lam H. (2005). Effects of Internal Curing Methods on Restrained Shrinkage and Permeability. Master’s Thesis.

[98] Mechtcherine V., Dudziak L., Hempel S. (2009). Mitigating Early Age Shrinkage of Ultra-High Performance Concrete by Using Super Absorbent Polymers (SAP). Creep, Shrinkage and Durability Mechanics of Concrete and Concrete Structures, Proceedings of the 8th International Conference on Creep, Shrinkage and Durability Mechanics of Concrete and Concrete Structures, Ise-Shima, Japan, 30 September–2 October 2008.

[99] Larianovsky P. (2007). Internal Curing of Concrete Using Super-Absorbent Polymers.

[100] Mechtcherine V. (2006). Internal Curing by Super Absorbent Polymers (SAP)—Effects on Material Properties of Self-Compacting Fibre-Reinforced High Performance Concrete. Proceedings of the International RILEM Conference on Volume Changes of Hardening Concrete: Testing and Mitigation.

[101] Karthikeyan V., Sabari K., Sasikumar S., Seyatharasan S., Thirumoorthi S. (2018). Self-Curing Concrete by Using Super Absorbent Polymer. Int. J. Eng. Res. Technol. (IJERT).

[102] Hasholt M.T., Jespersen M., Jensen O. (2010). Mechanical Properties of Concrete with SAP. Part I: Development of Compressive Strength. International RILEM Conference on Use of Superabsorbent Polymers and Other New Additives in Concrete, Lyngby, Denmark, 15–18 August 2010.

[103] Mechtcherine V., Gorges M., Schroefl C., Assmann A., Brameshuber W., Ribeiro A.B., Cusson D., Custódio J., Da Silva E.F., Ichimiya K. (2014). Effect of Internal Curing by Using Superabsorbent Polymers (SAP) on Autogenous Shrinkage and Other Properties of a High-Performance Fine-Grained Concrete: Results of a RILEM Round-Robin Test. Mater. Struct..

[104] Shen D., Wang X., Cheng D., Zhang J., Jiang G. (2016). Effect of Internal Curing with Super Absorbent Polymers on Autogenous Shrinkage of Concrete at Early Age. Constr. Build. Mater..

[105] Zuo W., Feng P., Zhong P., Tian Q., Gao N., Wang Y., Yu C., Miao C. (2017). Effects of Novel Polymer-Type Shrinkage-Reducing Admixture on Early Age Autogenous Deformation of Cement Pastes. Cem. Concr. Res..

[106] Lesovik V., Popov D., Fediuk R., Glagolev E., Yoo D.-Y. (2020). Improvement of Mechanical and Durability Behaviors of Textile Concrete: Effect of Polymineral Composite Binders and Superabsorbent Polymers. J. Mater. Civil. Eng..

[107] Lefever G., Aggelis D.G., De Belie N., Raes M., Hauffman T., Van Hemelrijck D., Snoeck D. (2020). The Influence of Superabsorbent Polymers and Nanosilica on the Hydration Process and Microstructure of Cementitious Mixtures. Materials.

[108] Lefever G., Tsangouri E., Snoeck D., Aggelis D.G., De Belie N., Van Vlierberghe S., Van Hemelrijck D. (2020). Combined Use of Superabsorbent Polymers and Nanosilica for Reduction of Restrained Shrinkage and Strength Compensation in Cementitious Mortars. Constr. Build. Mater..

[109] Steyl L. (2016). Plastic Cracking of Concrete and the Effect of Depth. Ph.D. Thesis.

[110] Mindess S., Young J.F., Darwin D. (2003). Concrete.

[111] Friedrich S. (2012). Superabsorbent Polymers (SAP). Application of Super Absorbent Polymers (SAP) in Concrete Construction: State-of-the-Art Report Prepared by Technical Committee 225-SAP.

[112] Dudziak L., Mechtcherine V. (2010). Enhancing Early-Age Resistance to Cracking in High Strength Cement-Based Materials by Means of Internal Curing Using Super Absorbent Polymers. RILEM Proc. PRO.

[113] Meyer D.M., Boshoff W.P., Combrinck R. (2020). Utilising Super Absorbent Polymers as Alternative Method to Test Plastic Shrinkage Cracks in Concrete. Constr. Build. Mater..

[114] Cohen M.D., Mobasher B. (1988). Drying Shrinkage of Expansive Cements. J. Mater. Sci..

[115] Gribniak V., Kaklauskas G., Kliukas R., Jakubovskis R. (2013). Shrinkage Effect on Short-Term Deformation Behavior of Reinforced Concrete—When It Should Not Be Neglected. Mater. Des..

[116] Liu R., Xiao H., Li H., Sun L., Pi Z., Waqar G.Q., Du T., Yu L. (2018). Effects of Nano-SiO_2_ on the Permeability-Related Properties of Cement-Based Composites with Different Water/Cement Ratios. J. Mater. Sci..

[117] Hong G., Choi S. (2017). Rapid Self-Sealing of Cracks in Cementitious Materials Incorporating Superabsorbent Polymers. Constr. Build. Mater..

[118] Snoeck D., Malm F., Cnudde V., Grosse C.U., Van Tittelboom K. (2018). Validation of Self-Healing Properties of Construction Materials through Nondestructive and Minimal Invasive Testing. Adv. Mater. Interfaces.

[119] Snoeck D., Van den Heede P., Van Mullem T., De Belie N. (2018). Water Penetration through Cracks in Self-Healing Cementitious Materials with Superabsorbent Polymers Studied by Neutron Radiography. Cem. Concr. Res..

[120] Van Mullem T., De Brabandere L., Van de Voorde E., Snoeck D., De Belie N. (2024). Influence of Superabsorbent Polymers on the Chloride Ingress of Mortar Measured by Chloride Diffusion and a Quasi-Steady-State Migration Test. Cem. Concr. Compos..

[121] Reinhardt H.W., Assmann A. (2012). Effect of Superabsorbent Polymers on Durability of Concrete. Application of Super Absorbent Polymers (SAP) in Concrete Construction: State-of-the-Art Report Prepared by Technical Committee 225-SAP.

[122] Reinhardt H.W., Assmann A. (2009). Enhanced Durability of Concrete by Superabsorbent Polymers. Brittle Matrix Compos..

[123] Ouattara Coumoin C., Wang F., Yang J., Liu Z. (2019). Effect of SAP on Properties of High Performance Concrete under Marine Wetting and Drying Cycles. J. Wuhan. Univ. Technol. Mater. Sci. Ed..

[124] Ma X., Liu J., Wu Z., Shi C. (2017). Effects of SAP on the Properties and Pore Structure of High Performance Cement-Based Materials. Constr. Build. Mater..

[125] Gräf V.H., Düsseldorf H.G. (1986). Verfahren Zur Prüfung Der Durchlässigkeit von Mörtel und Beton Gegenüber Gasen und Wasser. Beton.

[126] Snoeck D., De Belie N. (2019). Autogenous Healing in Strain-Hardening Cementitious Materials with and without Superabsorbent Polymers: An 8-Year Study. Front. Mater..

[127] Su A., Wang Y., Wang R., Chu Y., Xu W., Tian Q., Yao S., Meng Q., Wang W. (2024). Effects of Superabsorbent Polymers on the Pore Structure and Coefficient of Thermal Expansion of Cementitious Materials. Case Stud. Constr. Mater..

[128] Ferrara L., Van Mullem T., Alonso M.C., Antonaci P., Borg R.P., Cuenca E., Jefferson A., Ng P.L., Peled A., Roig-Flores M. (2018). Experimental Characterization of the Self-Healing Capacity of Cement Based Materials and Its Effects on the Material Performance: A State of the Art Report by COST Action SARCOS WG2. Constr. Build. Mater..

[129] Snoeck D., Criel P. (2019). Voronoi Diagrams and Self-Healing Cementitious Materials: A Perfect Match. Adv. Cem. Res..

[130] Snoeck D., Moerkerke B., Mignon A., De Belie N. (2020). In-Situ Crosslinking of Superabsorbent Polymers as External Curing Layer Compared to Internal Curing to Mitigate Plastic Shrinkage. Constr. Build. Mater..

[131] Yang H., Liu J., Jia X., Zhou Y., Ji H. (2020). Influence of NaCl Concentrations on the Crack-Sealing Behavior of Superabsorbent Polymers in Cementitious Materials. Constr. Build. Mater..

[132] Snoeck D., Steuperaert S., Van Tittelboom K., Dubruel P., De Belie N. (2012). Visualization of Water Penetration in Cementitious Materials with Superabsorbent Polymers by Means of Neutron Radiography. Cem. Concr. Res..

[133] Tang C., Dong R., Tang Z., Long G., Ma G., Wang H., Huang Y. (2024). Effect of SAP on the Properties and Microstructure of Cement-Based Materials in the Low Humidity Environment. Case Stud. Constr. Mater..

[134] Tenório Filho J.R., de Araújo M.A.P.G., Mannekens E., De Belie N., Snoeck D. (2022). Alginate- and Sulfonate-Based Superabsorbent Polymers for Application in Cementitious Materials: Effects of Kinetics on Internal Curing and Other Properties. Cem. Concr. Res..

[135] Kanellopoulou I.A., Kartsonakis I.A., Charitidis C.A. (2021). The Effect of Superabsorbent Polymers on the Microstructure and Self-Healing Properties of Cementitious-Based Composite Materials. Appl. Sci..

[136] Gwon S., Ahn E., Shin M. (2019). Self-Healing of Modified Sulfur Composites with Calcium Sulfoaluminate Cement and Superabsorbent Polymer. Compos. B Eng..

[137] Park B., Choi Y.C. (2018). Self-Healing Capability of Cementitious Materials with Crystalline Admixtures and Super Absorbent Polymers (SAPs). Constr. Build. Mater..

[138] Li D., Chen B., Chen X., Fu B., Wei H., Xiang X. (2020). Synergetic Effect of Superabsorbent Polymer (SAP) and Crystalline Admixture (CA) on Mortar Macro-Crack Healing. Constr. Build. Mater..

[139] Lefever G., Snoeck D., Aggelis D.G., De Belie N., Van Vlierberghe S., Van Hemelrijck D. (2020). Evaluation of the Self-Healing Ability of Mortar Mixtures Containing Superabsorbent Polymers and Nanosilica. Materials.

[140] Kua H.W., Gupta S., Aday A.N., Srubar W.V. (2019). Biochar-Immobilized Bacteria and Superabsorbent Polymers Enable Self-Healing of Fiber-Reinforced Concrete after Multiple Damage Cycles. Cem. Concr. Compos..

[141] Bana R.S., Grover M., Singh D., Bamboriya S.D., Godara S., Kumar M., Kumar A., Sharma S., Shekhawat P.S., Lomte D. (2023). Enhanced Pearl Millet Yield Stability, Water Use Efficiency and Soil Microbial Activity Using Superabsorbent Polymers and Crop Residue Recycling across Diverse Ecologies. Eur. J. Agron..

[142] Chindasiriphan P., Subwilai N., Intarasoontron J., Nuaklong P., Jongvivatsakul P., Chompoorat T., Pungrasmi W., Likitlersuang S. (2024). Synergistic Effects of Microencapsulated Bacterial Spores and Superabsorbent Polymer on Self-Healing Performance in Mortar. Constr. Build. Mater..

[143] González D.C., Mena Á., Mínguez J., Vicente M.A. (2021). Influence of Air-Entraining Agent and Freeze-Thaw Action on Pore Structure in High-Strength Concrete by Using CT-Scan Technology. Cold Reg. Sci. Technol..

[144] Bentz D.P., Jensen O.M. (2004). Mitigation Strategies for Autogenous Shrinkage Cracking. Cem. Concr. Compos..

[145] Sarbapalli D., Dhabalia Y., Sarkar K., Bhattacharjee B. (2016). Application of SAP and PEG as Curing Agents for Ordinary Cement-Based Systems: Impact on the Early Age Properties of Paste and Mortar with Water-to-Cement Ratio of 0.4 and Above. Eur. J. Environ. Civil. Eng..

[146] Riyazi S., Kevern J.T., Mulheron M. (2017). Super Absorbent Polymers (SAPs) as Physical Air Entrainment in Cement Mortars. Constr. Build. Mater..

[147] Falikman V.R. (2020). Effect of SAP on the Freeze-Thaw Resistance of Concrete: Tests According to Russian Standards. RILEM Bookseries.

[148] Mechtcherine V., Schröfl C., Wyrzykowski M., Gorges M., Lura P., Cusson D., Margeson J., De Belie N., Snoeck D., Ichimiya K. (2017). Effect of Superabsorbent Polymers (SAP) on the Freeze–Thaw Resistance of Concrete: Results of a RILEM Interlaboratory Study. Mater. Struct..

[149] (2010). Guide to Hot Weather Concreting.

[150] Bentz D.P., Weiss W.J. (2011). Internal Curing: A 2010 State-of-the-Art. Review.

[151] ACI Committee 231 Report on Early-Age Cracking: Causes, Measurement, and Mitigation. https://www.concrete.org/publications/internationalconcreteabstractsportal/m/details/id/51663499.

[152] (2008). Internal Curing of High Performance Concrete: Laboratory and Field Experiences.

[153] Kovler K., Jensen O., RILEM Technical Committee (2007). Internal Curing of Concrete: State of the Art Report of RILEM Technical Committee TC 196-ICC—“Internal Curing of Concrete”.

[154] (2017). Standard Specification for Lightweight Aggregate for Internal Curing of Concrete.

[155] Northeast Solite Internal Curing Specification. https://northeastsolite.com/?page_id=418.

[156] ESCSI Guide Specifications for Internally Cured Concrete. https://www.utelite.com/wp-content/uploads/2018/06/ESCSIinternal.pdf.

[157] NYSDOT (2010). Specification 584.3101—18.

[158] (2015). Specific Gravity Factor and Absorption of Lightweight Fine Aggregate.

[159] Weiss W.J., Montanari L. (2017). Guide Specification for Internally Curing Concrete.

[160] (2008). Moisture Content of Lightweight Fine Aggregate.

[161] Mignon A., Snoeck D., Dubruel P., Van Vlierberghe S., De Belie N. (2017). Crack Mitigation in Concrete: Superabsorbent Polymers as Key to Success?. Materials.

[162] (2013). Concrete—Specification, Performance, Production and Conformity.

[163] (2020). Test Method for Determination of the Free Swell Capacity in Saline by Gravimetric Measurement.

[164] Mechtcherine V., Schroefl C. (2014). Proceedings of the International RILEM Conference on Application of Superabrsorbent Polymers and Their New Admixtures in Concrete Construction.

[165] Mechtcherine V., Schröfl C., Reichardt M., Klemm A.J., Khayat K.H. (2019). Recommendations of RILEM TC 260-RSC for Using Superabsorbent Polymers (SAP) for Improving Freeze–Thaw Resistance of Cement-Based Materials. Mater. Struct..

[166] Mechtcherine V., Reinhardt H.W. (2012). Application of Super Absorbent Polymers (SAP) in Concrete Construction: State-of-the-Art Report Prepared by Technical Committee 225-SAP.

[167] Zhu C., Li X., Xie Y. (2014). Influence of SAP on the Performance of Concrete and Its Application in Chinese Railway Construction. Application of Superabsorbent Polymers and Other New Admixtures in Concrete Construction, Proceedings of International Conference, RILEM Publications, Dordrecht, The Netherlands, 3–5 November 2014.

[168] Mechtcherine V. (2016). Use of Superabsorbent Polymers (SAP) as Concrete Additive. RILEM Tech. Lett..

[169] Liu J., Yu C., Shu X., Ran Q., Yang Y. (2019). Recent Advance of Chemical Admixtures in Concrete. Cem. Concr. Res..

[170] China Zun Tower Facts and Information The Tower Info 2017. https://thetowerinfo.com/buildings-list/china-zun-tower/.

[171] Zheng X., Han M., Liu L. (2021). Effect of Superabsorbent Polymer on the Mechanical Performance and Microstructure of Concrete. Materials.

[172] Kim I.S., Choi S.Y., Choi Y.S., Yang E.I. (2021). An Experimental Study on Absorptivity Measurement of Superabsorbent Polymers (SAP) and Effect of SAP on Freeze-Thaw Resistance in Mortar Specimen. Constr. Build. Mater..

[173] Xu Y., Yuan Q., De Schutter G., Wang F., Li H. (2023). Detecting the Damage of Concrete Subjected to Fatigue Load Coupled with Freeze-Thaw Cycles Using Alternating Current Electric Impedance Spectroscopy. Cem. Concr. Compos..

[174] Zhang H., Sarker P.K., Xiao L., Ai J., He B., Ren Q., Zhu X., Zhang Y. (2023). Durability of Low-Carbon Geopolymer Mortar: Different Responses to Cryogenic Attack Caused by Water Content and Freeze-Thaw Mediums. Cem. Concr. Compos..

[175] Brüdern A.-E., Mechtcherine V., Jensen O.M., Hasholt M.T., Laustsen S. (2010). Multifunctional Use of SAP in Strain-Hardening Cement-Based Composites. Proceedings of the International RILEM Conference on Use of Superabsorbent Polymers and Other New Additives in Concrete.

[176] Snashall H.T. (1991). Cementitious Mixes. South African Patent.

[177] Jensen O.M. (2000). Water-Entrained Cement-Based Materials. PCT Patent.

[178] Jensen O.M. (2008). Use of Superabsorbent Polymers in Construction Materials. Proceedings of the 1st International Conference on Microstructure Related Durability of Cementitious Composites.

[179] Tsubakimoto T., Shimomura T., Kobayashi H. (1987). Japan. Kokai Tokkyo Koho.

[180] Shimomura T., Namba T., Buchholz F.L., Peppas N.A. (1994). Superabsorbent Polymers: Science and Technology.

[181] Buchholz F.L., Graham A.T. (1998). Modern Superabsorbent Polymer Technology.

